# The Impact of Single Nucleotide Polymorphism in the Long Non-coding MEG3 Gene on MicroRNA-182 and MicroRNA-29 Expression Levels in the Development of Breast Cancer in Egyptian Women

**DOI:** 10.3389/fgene.2021.683809

**Published:** 2021-08-04

**Authors:** Olfat Shaker, Ghada Ayeldeen, Amr Abdelhamid

**Affiliations:** ^1^Medical Biochemistry and Molecular Biology Department, Kasr Al Ainy Hospital, Faculty of Medicine, Cairo University, Cairo, Egypt; ^2^Biochemistry Department, Faculty of Pharmacy, October University for Modern Sciences and Arts, 6th of October City, Egypt

**Keywords:** miRNAs, lncRNAs, MEG3, BC, SNP

## Abstract

Early-stage detection of BC is a critical factor for effective treatment of the disease and can increase the survival rate of BC patients. Long non-coding RNAs can act as miRNA decoys by sequestering miRNAs, thus acting as competing endogenous RNAs and leading to re-expression of miRNA target genes. Maternally expressed 3 (MEG3) is LncRNA and it was reported to be tumor suppressor in breast cancer. The study aims to investigate the effect of MEG3 SNP (rs7158663 G/A) and its association with breast cancer risk in the Egyptian population. In addition, demonstrate the consequence of the MEG3 polymorphism on the expression levels of MEG3, miR-182, and miRNA-29. MEG3 rs7158663 G/A was genotyped and serum MEG3, miRNA-182, and miRNA-29 were measured in 180 breast cancer, 120 FA, and 150 controls by the qPCR. Frequencies of MEG3 rs7158663 GA/AA genotype and A allele were significantly higher in BC patients compared to the controls results showed that serum MEG3 levels were significantly lower, according to the presence of the A allele in different study groups while the expression of miR-182 and miRNA 29 were significantly elevated. MEG3, miR-182, and miRNA-29 are key genes involved in the development of BC, are considered as a novel potential non-invasive diagnostic biomarker for BC.

## Introduction

The second most common lethal malignancy in women and the topmost cause of cancer-related death in women globally is breast cancer (BC) impacting around 2 million women per year and responsible for the majority of cancer-related deaths among women (Mathur et al., 2020). By 2050, the number of women diagnosed with breast cancer is expected to rise to 3.2 million (Momenimovahed and Salehiniya, 2019). BC is the most prevalent cancer among Egyptian women, accounting for 37.7% of the 13,000 new cases yearly (Nassar et al., 2020). Long non-coding RNAs (lncRNAs) are defined as RNAs longer than 200 nucleotides that are not translated into functional proteins. LncRNAs can affect drug toxicity and response in cancer patients by controlling gene expression at different levels: epigenetic, transcriptional, and post-transcriptional (Gutschner and Diederichs, 2012). It has been identified that lncRNAs play a role in carcinogenesis by controlling a variety of biological processes (Bhat et al., 2020).

LncRNA Maternally expressed 3 (MEG3) has been identified as a tumor suppressor in breast cancer (Bayarmaa et al., 2019). Via various pathways, MEG3 controls the expression of the p53 tumor suppressor gene and induces p53-dependent transcription (Ghafouri-Fard and Taheri, 2019). In many cancerous tissues, such as gastric cancer, colorectal cancer, ovarian cancer, and hepatocellular carcinoma, lncRNA MEG3 has been found to be downregulated (Ali et al., 2020). Single nucleotide polymorphisms (SNPs) have been considered a potential biomarker of genetic background to predict risk, progression, and treatment response to several diseases (Motawi et al., 2015). SNPs in MEG3 have been related to cell phenotypes, increased cancer risk, and chemotherapy toxicity in other cancers (Zheng et al., 2020).

Multiple cancers have been found to upregulate the microRNA-29 family. The abnormal expression of the miR-29 family is related to tumorigenesis and cancer progression, according to growing evidence (Wang Y. et al., 2013). Overexpression of miR-29b decreases apoptosis and inhibits the expression of the tumor suppressor phosphatase and tensin homolog (PTEN), resulting in increased tumor cell invasion and migration (Shaker et al., 2015). In patients with BC, miR-182-5 has been suggested to be ectopic (Zhang et al., 2017). Similarly, it has been found in BC that the pro-apoptosis and antiproliferation effects are activated by inhibition of miR-182-5p via regulating Caspase 9 expression (Zhao et al., 2019). However, the role of miR-182-5p in BC and its mechanism are not fully understood. Highly expressed miR-182 functions as a potential oncomir in BC (Moskwa et al., 2011).

Several reports have emphasized the critical interaction between lncRNAs and miRNAs in RNA regulation processes. LncRNAs can act as miRNA traps by sequestering miRNAs, so acting as competing endogenous RNAs and leading to re-expression of miRNA target genes (Ratti et al., 2020). Besides, lncRNAs can promote gene expression by competing with miRNAs for specific binding sites in the non-coding regions of mRNAs and preventing the transcriptional repression caused by miRNAs (Bhat et al., 2020). MEG3 was suggested to be a molecular sponge for miR-182, and miR-182 inhibition had similar effects as MEG3 overexpression (Liu et al., 2018). *In vitro*, deregulated expression of the lncRNA MEG3 in HCC was shown to have a functional impact, and a potential mechanism through which deregulated tissue-specific expression of miR-29a in HCC could epigenetically modulate MEG3 expression via promoter hypermethylation was established (Braconi et al., 2011).

However, there is no clear evidence to support the hypothesis that miR-182, miR-29, and MEG3 are related. The interaction between MEG3 as a lncRNA that can function as miRNA decoys by sequestering miRNA-182 and miRNA-29 was investigated in this report. The purpose of the study is to investigate the effect of MEG3 SNP (rs7158663 G/A) and its association with breast cancer risk. In addition, demonstrate the consequence of the MEG3 polymorphism on the expression levels of MEG3, miR-182, and miRNA-29 in the serum of BC patients in the Egyptian population revealing their diagnostic and prognostic role in the early detection of BC.

## Materials and Methods

### Subjects

A total of 450 Egyptian patients from the General Surgery Department, Kasr Al-Aini Hospital were enrolled in the current study. Patient history and clinical examination were used for the initial diagnosis. Mammography and surgical biopsies were used to confirm the diagnosis of the enrolled patients. Consequently, they were categorized into three main groups. Group I comprises the control group that was recruited during the routine checkup (150 age-matched healthy females; no family history of breast cancer or even fibroadenoma or palpable breast masses; no administered contraceptives; no hypertension or diabetes mellitus). Group II included 120 patients with fibroadenoma (FA). The clinical information was obtained from the reports of the patients including age, parity status, family history, contraceptive usage as well as the state of menstrual cycles. Group III included 180 patients suffering from breast cancer. They were newly diagnosed postmenopausal women with no history of breast cancer. Noticeably, all of them experienced the previous usage of oral contraceptives, also all of them with parity status more than two times. Before participation in the study, none of the patients had received chemotherapy, antihormonal, or radiotherapy treatment. Since the age, tumor size, tumor type, TNM staging (tumor size—the number of lymph nodes involved metastasis) are crucial matters in cancer, they were involved in this study. The status of estrogen and progesterone receptors (ER/PR status) was also determined; ER/PR+ve denotes ER/PR positivity, while ER/PR–ve denotes ER/PR negativity. The current study was approved by the ethical committee of the Faculty of Medicine, Cairo University. All the incorporated patients signed a written consent after the declaration of the general aim and procedure of the study. The ethical principles of Helsinki were respected and followed in this study.

### Methods

#### DNA Extraction and Genotyping

A QIA amplification extraction kit (Qiagen, Netherlands) was used to extract DNA from whole EDTA blood samples according to the manufacturer's instructions. The NanoDrop (ND)-1000 spectrophotometer was used to quantify and determine DNA purity (NanoDrop Technologies, Inc., USA). The purified DNA was used to genotype lncRNA MEG3 rs7158663 (G/A) [c 9693465 10, PN 4351379, Lot: P171206 012E08] in a real-time PCR with the Taq-Man allelic discrimination assay (Applied Biosystems, USA) using predesigned unique primer/probe sets for lncRNA MEG3 rs71586. A Rotor-gene Q Real-Time PCR System was used to perform real-time PCR (Qiagen, Valencia, CA, USA). PCR program was as follows: 95°C for 10 min for an initial denaturation, 45 cycles at 92°C for 15 s, followed by 60°C for 90 s for annealing and extension, and fluorescence was measured at the end of each cycle as well as the endpoint.

#### RNA Extraction

RNA with microRNAs was extracted from 200 μl serum using the miRNeasy extraction kit (Qiagen, Valencia, CA, USA), 1,000 μl QIAzol lysis reagent, and 5 min at room temperature. Chloroform (200 μl) was applied, vortexed for 15 s, and then incubated at room temperature for 2–3 min. After that, centrifugation at 12,000×*g* for 15 min at 4°C was performed. The upper watery phase was isolated and 1.5 times its volume was applied to ethanol (100%). Then 700 μl of this mixture was centrifuged (8,000×*g*) at RT for 15 s in an RNeasy Mini spin column in a collection tube (2 ml). After the mixture had passed through the column full, 700 μl of RWT buffer was applied to each column and centrifuged again (at 8,000×*g*; at RT) for 15 s. The column was loaded with 500 μl of buffer RPE (centrifuged; 8,000×*g*; RT; 15 s). The column was moved to a new 1.5-ml collection tube and 50 μl RNase-free water was applied to the column before centrifuging for 1 min at 8,000×*g* to elute RNA.

#### Reverse Transcription and Real-Time Quantitative PCR

Reverse transcription was performed on total RNA in a final volume of 20 μl RT reactions (incubated for 60 min at 37°C and 5 min at 95°C) according to the manufacturer's instructions using the themiScript II RT package (Qiagen, USA). A MiScript SYBR Green PCR kit (Qiagen, USA) and miScript primer assays miR-29b-2, miR-182-5p, and MEG3 were used for real-time qPCR (Qiagen, USA). In a total volume of 20 μl reaction, 20 ng of cDNA was used as a guide, with the following conditions: denaturation at 95°C for 15 min, followed by 40 cycles of 94°C for 15 s, 55°C for 30 s, and 70°C for 34 s, during which fluorescence was acquired and detected by Rotor-gene Q Real-time PCR system (Qiagen, USA). Melting curve analyses were performed after the PCR cycles to confirm the particular generation of the predicted PCR product. SNORD was used as an endogenous regulator because there is no known control miRNA in serum. Using the Ct process, the expression levels of miR-29b-2, miR-182-5p, and MEG3 were determined. The number of qPCR cycles needed for the fluorescent signal to cross a given threshold is known as the cycle threshold (Ct) value. Ct was determined by subtracting SNORD Ct values from target microRNA Ct values. ΔΔCt was calculated by subtracting the ΔCt of the control samples from the ΔCt of the cancer samples. The fold change in miR-29b, miR-182-5p, and MEG3 expression was calculated by equation 2^−Δ*ΔCt*^.

### Statistical Analysis

Data are presented as mean ± SEM, mean (95% CI), or number (percentage) when appropriate. Categorical data were compared by χ^2^ or Fisher's exact test when appropriate. Continuous variables were compared using Student's *t*-test or one-way ANOVA followed by Tukey's *post-hoc* test when appropriate. MEG3, miRNA-29, and miRNA-182 diagnostic accuracy was assessed using receiver-operating-characteristic (ROC) analysis, and the region under the curve (AUC) was measured. AUC <0.6 was deemed non-important, 0.7–0.89 was estimated a possible discriminator, and AUC >0.9 was deemed a significant discriminator. Statistical analyses were performed using GraphPad Prism 5.0 (GraphPad Software, CA).

## Results

### Characteristics of the Study Population

The baseline characteristics (demographic, clinical, and pathological) of the 180 BC, 120 FA cases, and 150 healthy controls are shown in Table 1. There was a significant difference between breast cancer and FA patients although there was no significant difference between controls and FA group as regard age (*p* < 0.0001) with the mean ages of 42.73 ± 8.91, 42.31 ± 12.16, and 52.54 ± 10.81 years for controls, FA, and BC cases, respectively. Eighty five percent (153 patients) show negative ER/PR status, while 15% (27 patients) show positive ER/PR status. The number of invasive ductal breast cancer patients was 165 (91.6%) and that of invasive lobular type subjects was 15 (8.4%). Regarding TNM staging, percentage of patients with T 2, 3, 4 were 62.2, 35.6, and 2.2%, respectively, whereas the percentage of patients with N1, 2, 3 were 18.3, 57.8, and 23.9%, respectively. All patients in the current study had no metastasis. By ultrasound (U/S) examination, 80.5% of BC cases had fatty liver. As for clinicopathological data of the FA patients, 75% had irregular menstrual cycles and 17.2% had regular menstruation, while 7.8% were postmenopausal. Moreover, 8.3% use contraceptive pills, 22.2% were with a history of FA (recurrent fibroadenoma), and 35.6% of FA patients were with parity more than two times.

**Table 1:** Pathological and clinical data of the studied groups.

**Variables**	**Healthy controls**	**FA**	**BC**
	**(*n* = 150)**	**(*n* = 120)**	**(*n* = 180)**
Age (years)	42.73 ± 8.91	42.31 ± 12.16	52.54^*^± 10.81
**ER/PR status**			
Negative			153 (85%)
Positive			27 (15%)
**Tumor type**			
Invasive ductal			165 (91.6%)
Invasive lobular			15 (8.4%)
**TNM STAGING**			
**Tumor staging:**			
Stage 2			112 (62.2%)
Stage 3			64 (35.6%)
Stage 4			4 (2.2%)
**Nodes staging:**			
Stage 1			33 (18.3%)
Stage 2			104 (57.8%)
Stage 3			43 (23.9%)
Metastasis:			Zero
**Tumor size**			
<5 cm			105 (58.3%)
>5 cm			75 (41.7%)
**U/S**			
Normal			35 (19.5%)
Fatty liver			145 (80.5%)
**Menstruation**			
Premenopausal (Regular)		31 (17.2 %)	
Premenopausal (Irregular)		135 (75%)	
Postmenopausal		14 (7.8%)	180 (100%)
**Contraceptive use**			
No		165 (91.7%)	
Yes		15 (8.3%)	180 (100%)
**Family history**			
No		140 (77.8%)	180 (100%)
Yes		40 (22.2%)	
**Parity**			
≤2		116 (64.4%)	
>2		64 (35.6%)	180 (100%)

*BC, Breast Cancer; FA, Fibroadenoma; ER/PR, Estrogen Receptor/Progesterone Receptor; U/S, Ultrasound*.

* *Significant difference from the control group*.

### Genotypes and Allele Distribution of MEG3 Gene SNP Rs7158663 G/A

The observed genotype distributions of MEG3 rs7158663 G/A SNP agreed with those expected from Hardy–Weinberg equilibrium in all study groups. Results presented in Table 2 indicated that the frequencies of MEG3 rs7158663 GA/AA genotype and A allele were significantly higher in BC patients than controls (65% vs. 38%, χ^2^ = 25.16, *p* ≤ 0.0001 and 72.8% vs. 30%, χ^2^_=_ 120.71, *p* ≤ 0.0001, respectively).

**Table 2:** Differences in allele distribution and genotype frequency of MEG3 gene single nucleotide polymorphism (SNP) rs7158663 G/A between control, fibroadenoma, and Breast cancer groups.

**Groups**	***n***	**Genotype frequency**		**OR**	**95% CI**	**Allele frequency**		**OR**	**95% CI**
		**GG**	**GA + AA**			**G**	**A**		
Control	150	93 (62%)	57 (38%)			210 (70%)	90 (30%)		
Fibroadenoma	120	64 (53.3%)	56 (46.7%)	1.428	0.87–2.32	108 (45%)	132 (55%)	2.85	2.01–4.06
Breast cancer	180	63 (35%)	117 (65%)	3.03	1.93–4.75	98 (27.2%)	262 (72.8%)	6.23	4.44–8.75
		*X*^2^ = 25.16, *P* < 0.0001				*X*^2^ = 120.71, *P* < 0.0001			

*OR, odds ratio; 95% CI, 95% confidence interval*.

*Results are expressed as number and in parentheses percent. P < 0.05 is considered statistically significant after adjusting for age, sex, and BMI. Chi squared (X^2^) test was performed to compare categorical data*.

### Association Between MEG3 Polymorphisms With BC

As indicated in Table 2, subjects with the GA/AA genotype and A allele were at increased risk for BC (OR = 3.03, CI = 1.93–4.75, *p* ≤ 0.01 and OR = 6.23, CI = 4.44–8.75, *p* ≤ 0.01, respectively) compared with those having the GG genotype and G allele.

### Serum Levels of MEG3, miRNA-29, and miRNA-182

**Figure 1:**
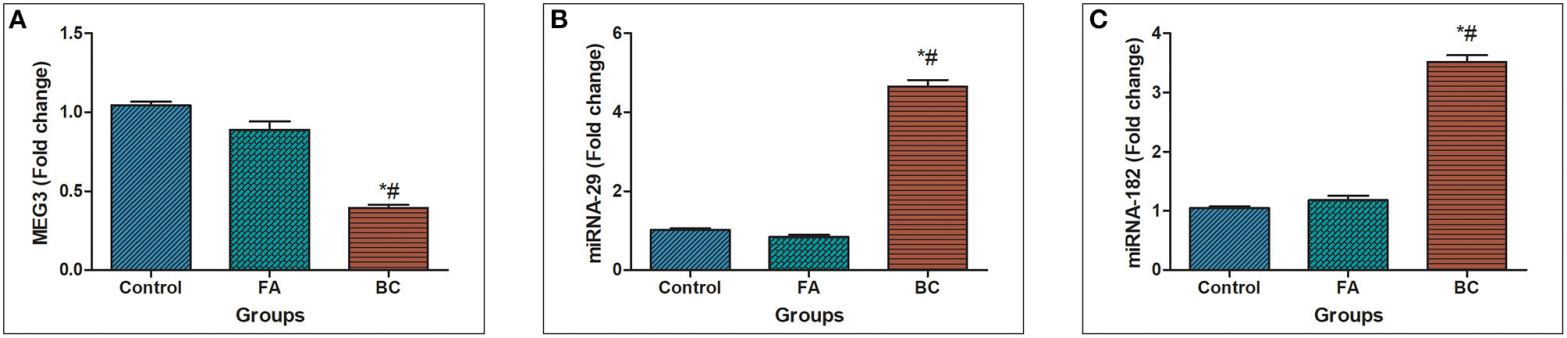
MEG3, miRNA-29, and miRNA182 expression levels in serum. **(A)** Fold change in serum expression MEG3 level in patients with BC (*n* = 180) and fibroadenoma (*n* = 120) relative to healthy subjects (*n* = 150). **(B)** Fold change in serum expression miRNA-29 level in patients with BC (*n* = 180) and fibroadenoma (*n* = 120) compared with healthy subjects (*n* = 150). Values are expressed as mean ± SEM (95% CI). **(C)** Fold change of serum expression miRNA-182 level in patients with BC (*n* = 180) and fibroadenoma (*n* = 120) compared with healthy subjects (*n* = 150). *Significant difference from the control group at *p* < 0.05. ^#^Significant difference from FA group at *p* < 0.05.

In BC subject's serum, MEG3 was downregulated significantly with a mean fold change of 0.393 ± 0.0219 (*p* < 0.005) in comparison with healthy subjects (Figure 1A). In addition, MEG3 serum levels in the BC group were significantly lower compared with the FA group with fold change 0.891 ± 0.0514 (*p* < 0.005). In BC subject's serum, miRNA-29 was upregulated significantly with a mean fold change of 4.65 ± 0.174 (*p* < 0.005) in comparison with the FA group with fold change 0.8413 ± 0.0518 (*p* < 0.005) and healthy subjects (Figure 1B). Moreover, In BC subject's serum, miRNA-182 was upregulated significantly with a mean fold change of 3.522 ± 0.1128 (*p* < 0.005) in comparison with the FA group with fold change 1.181 ± 0.07489 (*p* < 0.005) and healthy subjects (Figure 1C).

### The Relation Between MEG3 Gene SNP Rs7158663 G/A and Serum MEG3 Levels

According to the presence of the A allele in different study groups, serum MEG3 levels were significantly lower, as shown in Table 3. The serum level of MEG3 in patients with the GA+AA genotype (*n* = 117) in the BC group was significantly lower than in those with the GG genotype (*n* = 63) (*t* = 2.487, *p* = 0.037), according to the findings. Likewise, the serum MEG3 level of GA+AA genotype carriers in the fibroadenoma group (*n* = 56) was significantly lower than that of the GG genotype (*n* = 64) (*t* = 2.803, *p* = 0.0231). Also, there is no significant difference between GA+AA genotype carriers and GG genotype carriers in the control group (*t* = 1.452, *p* = 0.168).

**Table 3:** Differences in expression levels of MEG3 among different genotype carriers of MEG3 gene rs7158663 (GG and GA + AA) in study groups.

**Groups**	**Genotype**	***n***	**MEG3 [Fold change 2(ΔΔCT)]**
Control	GG	93	1.19 ± 0.041
	GA + AA	57	1.12 ± 0.038
Fibroadenoma	GG	64	0.92 ± 0.049
	GA + AA	56	0.72^*^± 0.037
Breast cancer	GG	63	0.52 ± 0.032
	GA + AA	117	0.36^*^± 0.021

*Data are given as mean ± SEM*,

* *Significant difference from GG genotype at P < 0.05*.

### Diagnostic Performance of Serum MEG3, miRNA-29, and miRNA-182

**Figure 2:**
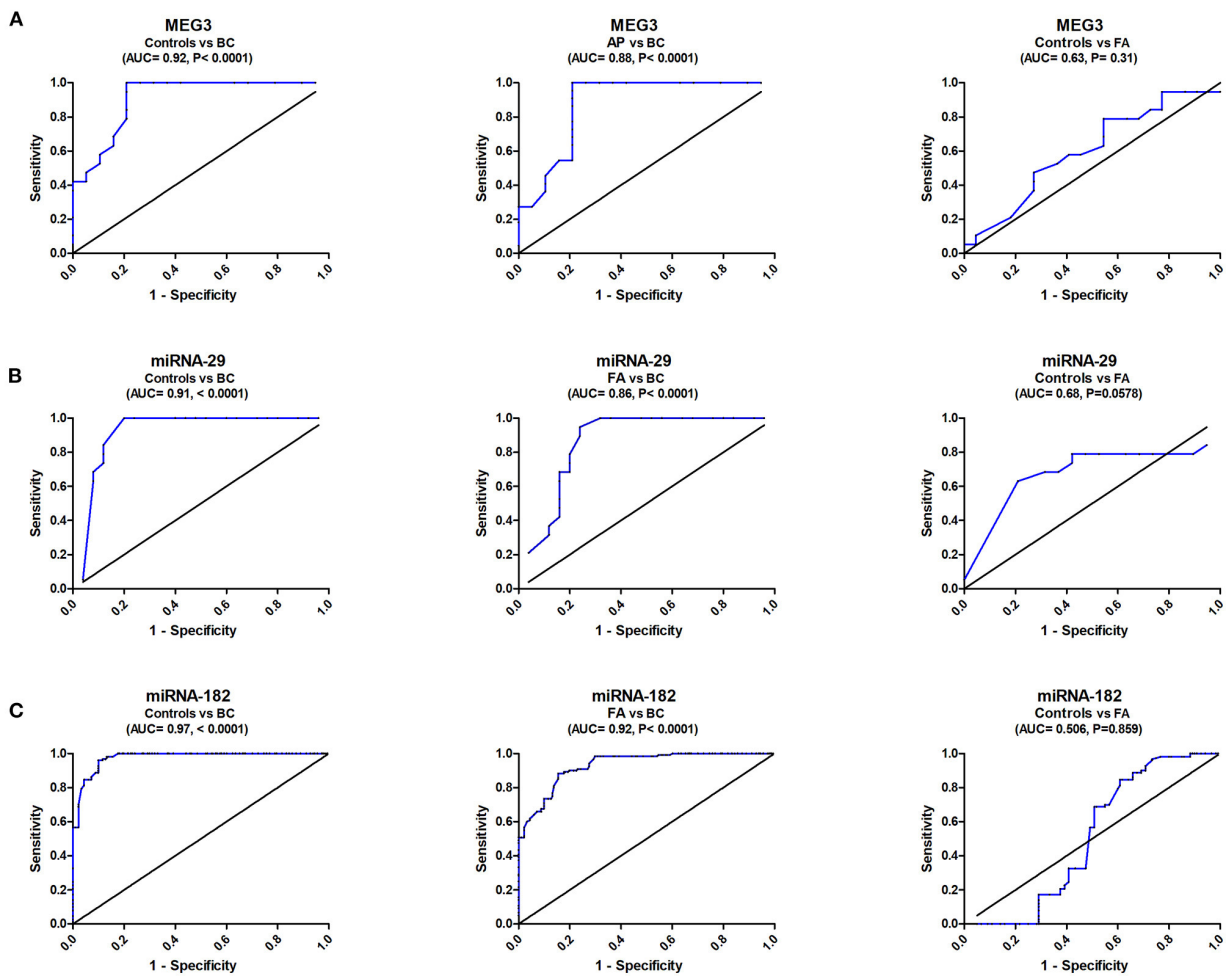
ROC curve study of serum MEG3, miRNA-29, and miRNA182 to differentiate the studied types, BC (*n* = 180) and FA (*n* = 120), from controls (*n* = 150). **(A)** Diagnostic accuracy of serum MEG3, **(B)** Diagnostic accuracy of serum miRNA-29, and **(C)** Diagnostic accuracy of serum miRNA182.

ROC analysis revealed that serum MEG3 discriminated healthy controls from BC with AUC = 0.92, 95% CI = 0.823–1.05, *p* < 0.0001, with sensitivity = 100%, specificity = 79.1%, and at a cutoff >0.43-fold. Serum MEG3 distinguished controls from FA patients with AUC = 0.63, 95% CI = 0.431–0.801, *p* = 0.301, with sensitivity = 84.2%, specificity = 27.3%, and at a cutoff >0.66-fold. In addition, serum MEG3 discriminated FA from BC with AUC = 0.88, 95% CI = 0.762–0.981, *p* < 0.0001, with sensitivity = 95.1%, specificity = 78.3%, and at a cutoff >0.46-fold (Figure 2A).

ROC analysis also revealed that serum miRNA-29 discriminated healthy controls from BC with AUC = 0.916, 95% CI = 0.823–1.012, *p* < 0.0001, with sensitivity = 100%, specificity = 80%, and at a cutoff <2.32-fold. Serum miRNA-29 also distinguished controls from FA patients with AUC = 0.68, 95% CI = 0.4976–0.8625, *p* = 0.0578, with sensitivity = 62.9%, specificity = 77.8%, and at a cutoff <1.15-fold. It also distinguished FA from BC with AUC = 0.86, 95% CI = 0.745–0.975, *p* < 0.0001, with sensitivity = 94.4%, specificity = 76%, and at a cutoff <1.96-fold (Figure 2B).

ROC analysis similarly showed that serum miRNA-182 discriminated healthy controls from BC with AUC = 0.97, 95% CI = 0.927–1.009, *p* < 0.0001, with sensitivity = 98%, specificity = 86.7%, and at a cutoff <1.85-fold. Serum miRNA-182 also distinguished controls from FA patients with AUC = 0.506, 95% CI = 0.428–0.842, *p* = 0.0578, with sensitivity = 70%, specificity = 45%, and at a cutoff <1.15-fold. It also distinguished FA from BC with AUC = 0.92, 95% CI = 0.791–0.9473, *p* < 0.0001, with sensitivity = 90%, specificity = 80%, and at a cutoff <2.36-fold (Figure 2C).

## Discussion

Based on the published studies, the incidence rate of breast cancer varies greatly with race and ethnicity and is higher in developed countries, so design and implementation of screening programs seem critical (Momenimovahed and Salehiniya, 2019). LncRNAs have arisen as critical regulators of cellular processes, and abnormal lncRNA levels can be vital cancer-specific diagnostic markers (Shaker et al., 2020; Nandwani et al., 2021). MEG3 was highlighted as a tumor suppressor lncRNA in BC tissues; abnormally expressed MEG3 can be distinguished in serum (Zhu et al., 2019). SNP function prediction exhibited that differences in genotype frequency and allele distribution of MEG3 gene SNP rs7158663 G/A might affect transcription factor binding sites (Han et al., 2018). Consequently, based on the evidence mentioned previously, we proposed a hypothesis that the polymorphism of lncRNA MEG3 may be a regulatory SNP and contribute to the risk of BC through altering MEG3 serum expression level. Besides, exploring the effect of MEG3 SNP (rs7158663 G/A) and its association with breast cancer risk in addition to its modulatory effect in expression levels of miR-182 and miRNA-29 in the serum of BC patients in the Egyptian women reveals their diagnostic and prognostic role in the early detection of BC.

MEG3 was found to be substantially downregulated in BC patients relative to healthy controls and fibroadenoma patients in the current study. These findings are consistent with previous findings which confirmed that MEG3 expression was found to be lower in clinical BC tissues compared with adjacent normal tissues, and MEG3 expression was found to be linked to differentiation grade, TNM stage, and lymph node metastasis in BC (Zhang et al., 2016; Mathias et al., 2019; Dong et al., 2021). Previous research reported dysregulated MEG3 expression in many human malignancies, and MEG3 functions as a tumor suppressor (He et al., 2017). By targeting Bcl-2 in prostate cancer cells, Yao Shi discovered that MEG3 played a critical role in controlling cell proliferation, apoptosis, and migration (Shi et al., 2018). MEG3 could be a bad prognostic biomarker in BC, according to Sun et al. (2014).

MEG3 polymorphisms have been linked to cancer risk and therapeutic response in cancer patients (Ghafouri-Fard and Taheri, 2019). MEG3 rs7158663 AA genotype confers risk of colorectal cancer in the Chinese population, according to Cao et al. (2016) which is similar to the findings of the current research, which showed that the frequencies of MEG3 rs7158663 GA/AA genotype and A allele were significantly higher in BC patients compared with controls. Furthermore, subjects with the GA/AA genotype and A allele were more likely to develop BC than those with the GG genotype and G allele. According to the RNAsnp forecast, rs7158663 in MEG3 altered the folding structures of MEG3 (Cao et al., 2016). As a result, we hypothesize that rs7158663 is a regulatory SNP that controls MEG3 expression and contributes to breast cancer genetic susceptibility (Zheng et al., 2020). The results of the current study showed that serum MEG3 levels were significantly lower depending on the presence of the A allele in different study groups among different genotype carriers of the MEG3 gene rs7158663 (GG and GA+AA). The serum level of MEG3 in patients with GA+AA genotype carriers in the BC group was significantly lower than in those with the GG genotype, according to the current report. In the fibroadenoma patients, the serum MEG3 level of GA+AA genotype carriers was significantly lower than that of the GG genotype. Besides, there is no significant difference between GA+AA genotype carriers and GG genotype carriers in the control group.

However, a growing number of studies have shown that microRNAs have a critical role in the pathogenesis of many diseases (Zaafan and Abdelhamid, 2021). The miR-29 family is downregulated in a variety of cancers and that it can suppress tumors such as breast, bladder, and pancreatic cancer (Kwon et al., 2016, 2019; Wang et al., 2018). The current study found significantly higher serum miRNA-29b levels in BC patients relative to safe controls, which is reliable with a previous study that found mir-29b was upregulated in BC cases compared with controls (Shaker et al., 2015). Overexpression of miR-29b inhibits the expression of the phosphatase and tensin homolog (PTEN) tumor suppressor, impairing apoptosis and increasing tumor cell migration and invasion, according to previous studies (Shaker et al., 2015; Liu et al., 2019; Zhong et al., 2019). Recently, several studies support that miR-182 acts as an oncogene in the development of BC (Kwon et al., 2016; Murugesan and Premkumar, 2021). MiR-182 is overexpressed in human BC tissues and cell lines (Wang et al., 2017). Our result showed overexpression of miRNA 182 in BC in comparison with the healthy control group. Overexpression of miR-182 inhibits DNA repair and decreases BRCA1 protein levels, while antagonizing miR-182 increases BRCA1 levels and causes resistance to the poly (ADP-ribose) polymerase 1 inhibitor (Wang P. Y. et al., 2013).

The ROC curve was created to examine MEG3's diagnostic ability, yielding a diagnostic accuracy (AUC = 90%) with a sensitivity of 100% in separating BC cases from control subjects. It had a promising prognostic value in BC staging because of its high ability to distinguish between FA and BC (AUC = 88%) and sensitivity of 95.1%. MicroRNA-29 had a high diagnostic accuracy (AUC = 91.6%) and sensitivity (100%) for control subjects from BC patients. It demonstrated a commendable ability to distinguish between FA and BC (AUC = 86%) and a sensitivity of 94.44%. MicroRNA-182 had a high diagnostic accuracy (AUC = 97%) and sensitivity (98%) for control subjects from BC patients. It demonstrated a remarkable ability to distinguish between FA and BC (AUC = 92%) with a sensitivity of 90%.

Both miRNAs and lncRNAs can influence tumor growth, invasion, and metastasis by increasing the activation of oncogenic pathways and restricting the expression of tumor suppressors, thereby influencing tumor growth, invasion, and metastasis. LncRNAs can counteract miRNAs by sequestering them, which is one of their many features (miRNA sponges) (Ratti et al., 2020). Long non-coding RNA MEG3 (tumor suppressors) was observed to participate in the silencing of miR-182 and miRNA 29. Inconsistent with the present study, the serum MEG3 expression was significantly down-expressed in BC patients; as a result, it was revealed that the expressions of miR-182 and miRNA 29 were significantly elevated and had a positive correlation with the degree of malignancy.

## In Summary

Early-stage detection of BC is a critical factor for effective treatment of the disease and can increase the survival rate of BC patients. Our findings open the door to the possibility of MEG3, miRNA-29b, and miRNA-182 being used as a diagnostic and prognostic biomarker in the clinical application. This research shows that frequencies of MEG3 rs7158663 GA/AA genotype and A allele were significantly higher in BC patients compared with controls. Also, MEG3 rs7158663 G/A mutant A allele is correlated to lower serum MEG3 expression levels. The current study elucidates the interaction between MEG3 as a long non-coding RNA that can act as miRNA decoys by sequestering miRNA-182 and miRNA-29. Serum MEG3 expression is significantly down-expressed in BC patients; as a consequence, the expression of miR-182 and miRNA 29 are overexpressed. In conclusion, the lncRNA (MEG3) and miRNAs (miR-182 and miRNA-29) are being investigated as a new non-invasive diagnostic biomarker for BC.

## Data Availability Statement

The raw data supporting the conclusions of this article will be made available by the authors, without undue reservation.

## Ethics Statement

The studies involving human participants were reviewed and approved by Faculty of Medicine Cairo University, Research Ethics Committee. The patients/participants provided their written informed consent to participate in this study.

## Author Contributions

OS and AA conceived and designed the research. AA and GA conducted experiments and analyzed data. AA wrote the manuscript with support from OS. All authors read and approved the manuscript.

## Conflict of Interest

The authors declare that the research was conducted in the absence of any commercial or financial relationships that could be construed as a potential conflict of interest.

## Publisher's Note

All claims expressed in this article are solely those of the authors and do not necessarily represent those of their affiliated organizations, or those of the publisher, the editors and the reviewers. Any product that may be evaluated in this article, or claim that may be made by its manufacturer, is not guaranteed or endorsed by the publisher.
